# Observational Cohort Study of Oral Mycobiome and Interkingdom Interactions over the Course of Induction Therapy for Leukemia

**DOI:** 10.1128/mSphere.00048-20

**Published:** 2020-04-15

**Authors:** Sarah Robinson, Christine B. Peterson, Pranoti Sahasrabhojane, Nadim J. Ajami, Samuel A. Shelburne, Dimitrios P. Kontoyiannis, Jessica R. Galloway-Peña

**Affiliations:** a Department of Statistics, Rice University, Houston, Texas, USA; b Department of Biostatistics, The University of Texas MD Anderson Cancer Center, Houston, Texas, USA; c Department of Infectious Diseases, Infection Control, and Employee Health, The University of Texas MD Anderson Cancer Center, Houston, Texas, USA; d Department of Genomic Medicine, The University of Texas MD Anderson Cancer Center, Houston, Texas, USA; Hackensack Meridian Health Center for Discovery and Innovation

**Keywords:** mycobiome, interkingdom interactions, leukemia, induction chemotherapy, *Malassezia*

## Abstract

Although the term “microbiome” refers to all microorganisms, the majority of microbiome studies focus on the bacteriome. Here, we characterize the oral mycobiome, including mycobiome-bacteriome interactions, in the setting of remission-induction chemotherapy (RIC) for acute myeloid leukemia (AML). Oral samples (*n* = 299) were prospectively collected twice weekly from 39 AML patients during RIC until neutrophil recovery. Illumina MiSeq 16S rRNA gene (V4) and internal transcribed spacer 2 (ITS2) sequencing were used to determine bacterial and fungal diversity and community composition. Intrakingdom and interkingdom network connectivity at baseline (T1) and at midpoint (T3) and a later time point (T6) were assessed via SPIEC-EASI (sparse inverse covariance estimation for ecological association inference). In this exploratory study, mycobiome α-diversity was not significantly associated with antibiotic or antifungal receipt. However, postchemotherapy mycobiome α-diversity was lower in subjects receiving high-intensity chemotherapy. Additionally, greater decreases in *Malassezia* levels were seen over time among patients on high-intensity RIC compared to low-intensity RIC (*P* = 0.003). A significantly higher relative abundance of *Candida* was found among patients who had infection (*P* = 0.008), while a significantly higher relative abundance of *Fusarium* was found among patients who did not get an infection (*P* = 0.03). Analyses of intrakingdom and interkingdom relationships at T1, T3, and T6 indicated that interkingdom connectivity increased over the course of IC as bacterial α-diversity diminished. In (to our knowledge) the first longitudinal mycobiome study performed during AML RIC, we found that mycobiome-bacteriome interactions are highly dynamic. Our study data suggest that inclusion of mycobiome analysis in the design of microbiome studies may be necessary to optimally understand the ecological and functional role of microbial communities in clinical outcomes.

**IMPORTANCE** This report highlights the importance of longitudinal, parallel characterization of oral fungi and bacteria in order to better elucidate the dynamic changes in microbial community structure and interkingdom functional interactions during the injury of chemotherapy and antibiotic exposure as well as the clinical consequences of these interrelated alterations.

## Abstract (precis)

This report highlights the importance of longitudinal, parallel characterization of oral fungi and bacteria in order to better elucidate the dynamic changes in microbial community structure and interkingdom functional interactions during the injury of chemotherapy and antibiotic exposure as well as the clinical consequences of these interrelated alterations.

## INTRODUCTION

There is increasing appreciation that microbiome composition affects treatment-related complications during cytotoxic chemotherapy and hematopoietic stem cell transplantation (HSCT) in patients with hematologic malignancy, including infections, gastrointestinal (GI) toxicities, and the development of graft-versus-host disease (GvHD) (1–3). For example, several studies have established an association between the gut microbiota and HSCT outcomes, including transplant-related mortality, relapse rates, and overall survival (4, 5). Although the term “microbiome” technically refers to all microorganisms, nearly all studies to date have focused on the role of bacteria rather than fungi (6).

Although still markedly understudied compared to the bacteriome, there are increasing numbers of reports linking the commensal fungi, or the “mycobiome,” to the health of patients with hematologic malignancy. For example, researchers have found that patients colonized with *Candida* sp. developed significantly more grade II to IV acute GvHD and GI GvHD than noncolonized patients (7). We previously found both the mouth and gut to be dominated by Candida albicans and Candida glabrata prior to mucormycosis during induction remission chemotherapy (8). Similarly, patients with a baseline microbiome community dominated by *Candida* and aciduric bacteria were more likely to develop oral candidiasis during antineoplastic treatment (9). Recently, variation in mycobiome composition was linked to treatment-related toxicities after autologous HSCT for multiple myeloma (10). Specifically, the composition of the baseline oral mycobiome was associated with vomiting as well as culture-negative neutropenic fever, while the fungal community on day 7 posttransplantion was associated with the incidence and severity of mucositis.

Patients with hematologic malignancy receive multiple types of drugs that may impact both fungal and bacterial communities. There is some evidence that the oral and intestinal bacterial microbiota in humans may be altered by cytotoxic chemotherapy (11), although the effects on the fungal microbiota are less clear. It has been previously shown that cytotoxic and cisplatin compounds have direct anti-*Candida* activity and also inhibit filamentation/conidation (12). More recently, investigators demonstrated that the oral microbiome is disrupted by 5-fluorouracil (5-FU) and doxorubicin chemotherapy, but they did not observe major changes in the composition of the mycobiome (13). Moreover, mice receiving both 5-fluorouracil and C. albicans had a loss of bacterial diversity and showed endogenous overgrowth of organisms such as *Enterococcus* and S*tenotrophomonas* in the oral mucosa (14). In addition to chemotherapy, antimicrobial therapy, a very common practice in this patient population, impacts mycobiome composition. For example, it was observed that antibiotics with antianaerobic activity affected the oral fungal community composition posttransplantion in multiple myeloma patients (10). Also, bacteremia with fluconazole-resistant *Candida* has been associated with exposure to several different antimicrobials, including carbapenems, clindamycin, and colistin (15). It has long been known that some antibacterial agents promote fungal growth via indirect effects (16), many of which promote *Candida* colonization (17, 18).

Given that the members of the mycobiome interact with the bacteriome in ways that can be beneficial, neutral, or detrimental to the host (19), we aimed to determine the changes in the oral mycobiome and in fungus-bacterium relationships in the setting of leukemia remission-induction chemotherapy (RIC) given that these patients typically receive combined antibiotics, antifungals, and cytotoxic chemotherapy (7). Given the key role of *Candida* in the health of the oropharynx and because oral microaspiration is the most common pathological mechanism of bacterial pneumonia, the most severe infection during RIC in this patient population, we focused on the oral mycobiome and its connectivity with the oral bacteriome (20, 21).

## RESULTS AND DISCUSSION

### Longitudinal oral mycobiome characteristics of acute myeloid leukemia (AML) patients undergoing RIC from baseline until neutrophil recovery.

**FIG 1:**
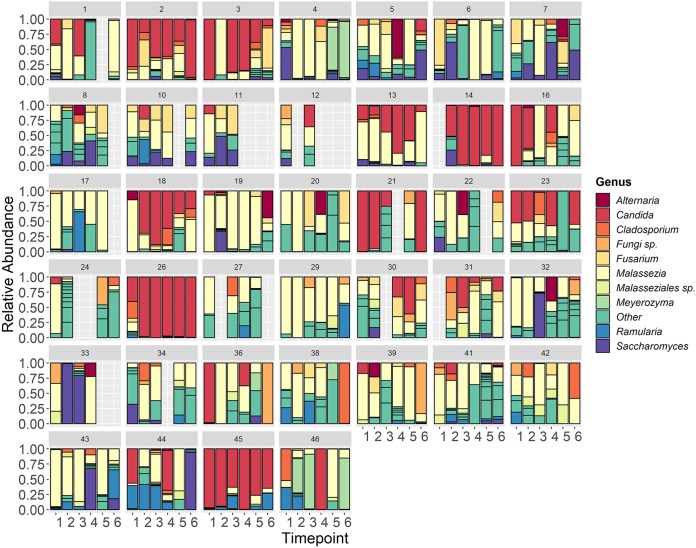
Abundances of top 10 fungal genera and in each patient across time. Stacked bar plots show the relative abundances of the 10 most abundant taxa identified to the genus level for each patient from baseline (T1) until the sixth sample collected (T6). Other, taxa not included in the 10 most abundant genera. *Fungi sp*, unknown/unidentified fungal genus.

**FIG 2:**
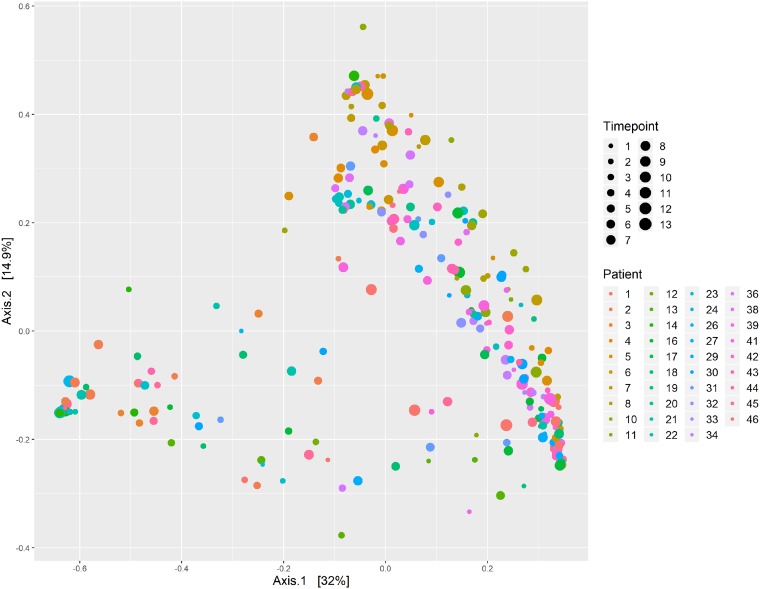
Mycobiome β-diversity over time. Results of principal-coordinate analysis (PCoA) of ITS2 OTUs via Bray-Curtis dissimilarity are presented. All patients are represented by distinctly colored data according to the inlaid figure legend. Increasing longitudinal durations per patient from baseline until neutrophil recovery are represented using increasingly larger circles.

**FIG 3:**
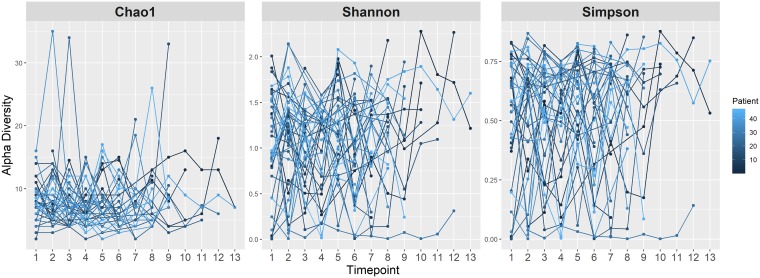
Mycobiome α-diversity over time. Mixed-effects models were used to plot the α-diversity metrics of Chao1, Shannon index, and Simpson index data over time for each patient (colored dark blue to light blue). Time point data represent the sequential time points at which samples were collected from baseline until neutrophil recovery approximately every 96 h.

Patient demographic and clinical data are shown in Table 1 and include approximately equal numbers of males (51%) and females (49%), with a mean age of 54 years. At the genus level, the most abundant genus observed across all time points was *Malassezia*, followed by *Candida*, *Saccharomyces*, *Fusarium*, and *Cladosporium*. This is very similar to the core set of oral mycobiome taxa previously identified among healthy individuals, which includes *Candida*, *Cladosporium*, Saccharomycetales, *Aspergillus*, *Fusarium*, *Cryptococcus*, *and Malassezia* (22, 23). Although *Aspergillus* was seen in at least one oral sample in over 70% of the patients, it had a detectable abundance in only 13% of all longitudinal samples. Additionally, among the samples which had a detectable abundance of *Aspergillus*, the median relative abundance was only 1.8% (interquartile range [IQR], 0.03% to 8.4%). This observation was interesting given that *Aspergillus* is the primary cause of invasive mold infection among patients with hematologic malignancy (24). The abundances of the top ten most abundant genera showed significant intrapatient and interpatient variability across time (Fig. 1). However, the β-diversity appeared to be more homogeneous within each patient across time, with the mean intrapatient Bray-Curtis dissimilarity between samples within the same patient being 0.649 among the cohort members versus an average interpatient β-diversity value of 0.745 for all samples across time points. This is consistent with another study that analyzed the oral mycobiome stability over 30 days, which revealed high interindividual diversity and yet indicated intraindividual stability (25). Using the results of principal-coordinate analysis (PCoA) shown in Fig. 2, we found no distinct grouping by patient or time point based on Bray-Curtis dissimilarity. We next sought to describe the oral mycobiome changes over the course of RIC. Through mixed-effects models, we found that mycobiome α-diversity did not significantly change over time (Chao1, *P* = 0.4639; Shannon, *P* = 0.8049; Simpson, *P* = 0.6637) (Fig. 3), unlike what has been previously been noted for the bacteriome, where diversity decreased from baseline until neutrophil recovery among leukemia patients receiving RIC (2, 26).

**TABLE 1:** Patient demographics and clinical characteristics

Characteristic	Values^*a*^
Demographics	
Mean age in yrs (range)	54 (23–84)
Male	20 (51)
Female	19 (49)

Chemotherapy intensity	
High	28 (72)
Low	11 (28)

Chemotherapy type	
Fludarabine-containing regimen^*b*^	17 (44)
Nonfludarabine high-intensity regimen^*c*^	11 (28)
Hypomethylator^*d*^	5 (13)
Other^*e*^	6 (15)

Infection	21 (54)
Microbiologically defined	9 (23)
Bacteremia	4 (10)
Urinary tract infection	3 (8)
Other	2 (5)
Clinically defined	12 (31)
Cellulitis	5 (13)
Pneumonia	3 (8)
Other	4 (10)

Antimicrobial administration	
Antifungals	39 (100)
Triazoles	31 (79)
Echinocandins	30 (77)
Amphotericin B	6 (15)
Broad-spectrum antibiotics	34 (87)
Carbapenem	26 (67)
Piperacillin-tazobactam	11 (28)
Cephalosporin	17 (44)

a Values represent number (percent) of patients except where otherwise indicated.

b Includes FIA and FLAG-Ida-based regimens.

c Includes CIA, IA, or CLIA-based regimens.

d Includes azacitidine or decitabine-based regimens.

e Includes LDAC-based regimens.

### Mycobiome characteristics correlate with chemotherapy subtype and antifungal administration.

**FIG 4:**
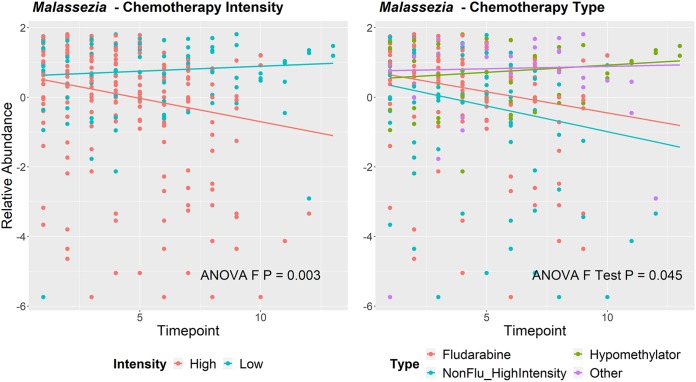
Changes in *Malassezia* over time stratified by chemotherapy intensity and chemotherapy type. Centered log ratio (clr) data represent transformed relative abundances of *Malassezia* with fitted lines from the mixed-effects model over time according to each variable of interest. *P* value data were derived from ANOVA F-test results. Panel A has fitted lines representing the intensities of the chemotherapy regimens, with high-intensity regimens indicated in coral and low-intensity regimens in aqua. Panel B further dissects these by chemotherapy subtype, with high-intensity fludarabine-containing regimens indicated in coral, hypomethylator-based therapies in green, non-fludarabine-containing high-intensity regimens in aqua, and other low-intensity/low-dose cytarabine-based therapies in purple.

We next sought to determine if mycobiome profiles were associated with clinical variables among the patients in the cohort, to include chemotherapeutic or antimicrobial administration. In this sampling of patients, 72% were on high-intensity chemotherapy, while 28% received low-intensity chemotherapy (Table 1). The majority of patients (44%) received fludarabine-based regimens, with the majority of the regimens being FIA (idarubicin, cytarabine, and fludarabine) or FLAG-Ida (fludarabine, idarubicin, and granulocyte-colony-stimulating factor). Twenty-eight percent of patients received nonfludarabine high-intensity regimens, which typically included CIA (idarubicin, cytarabine, and clofarabine)-based, IA (idarubicin and cytarabine)-based, or CLIA (cladribine, idarubicin, and cytarabine)-based regimens. Patients also receive hypomethylator-based treatments (such as decitabine or azacitadine) (13%) or other low-dose therapies to include LDAC (low-dose antimetabolite cytarabine)-based regimens (14%). Given the uneven sampling time (time to neutrophil recovery) among patients, we evaluated the mycobiome structure and composition at the baseline (T1), a midpoint (T3), and a later time point (T6), common among most patients. At T6, patients who received low-intensity RIC had significantly higher mycobiome α-diversity than patients who received high-intensity RIC (observed operational taxonomic units [OTUs], *P* = 0.02; Shannon, *P* = 0.054) (see Fig. S1 in the supplemental material). Although Kruskal-Wallis tests indicated that the relative abundances of the five most abundant fungal genera did not significantly differ between the four different chemotherapy subtypes at any single time point (see Table S1 in the supplemental material), we did determine chemotherapy subtypes to be significantly associated with the relative abundance of specific taxa over time. Specifically, patients who received high-intensity chemotherapies experienced a decrease in *Malassezia* abundance over time (*P* = 0.003, Fig. 4). This effect was not seen in other taxa analyzed (data not shown). To our knowledge, the changes in the oral mycobiome occurring as a result of chemotherapy have been studied only in regard to their role in chemotherapy-induced mucositis in the setting of 5-fluorouracil (5-FU) or doxorubicin-based chemotherapy in mice and humans (13, 14). In patients, although the authors expected chemotherapy to affect the oral mycobiome, no major changes in the composition of fungal communities were observed (13). Thus, our study would be the first to show specific changes in the oral mycobiome associated with chemotherapeutic intensity and type.

10.1128/mSphere.00048-20.1 FIG S1 Differences in mycobiome α-diversity (T6) based on chemotherapy intensity. A box plot compares observed OTUs and Shannon diversity at T6 between those who received high-intensity and low-intensity chemotherapy regimens. The *P* values are derived from a Mann-Whitney test. Download FIG S1, JPG file, 0.1 MB. Copyright © 2020 Robinson et al. 2020 Robinson et al. This content is distributed under the terms of the Creative Commons Attribution 4.0 International license.

10.1128/mSphere.00048-20.4 TABLE S1 Taxon abundance differences by chemotherapy subtype. *P* values represent results of Kruskal-Wallis test performed with data from each time point. Listed are the top 5 taxa by abundance across all 6 time points. Download Table S1, DOCX file, 0.02 MB. Copyright © 2020 Robinson et al. 2020 Robinson et al. This content is distributed under the terms of the Creative Commons Attribution 4.0 International license.

**FIG 5:**
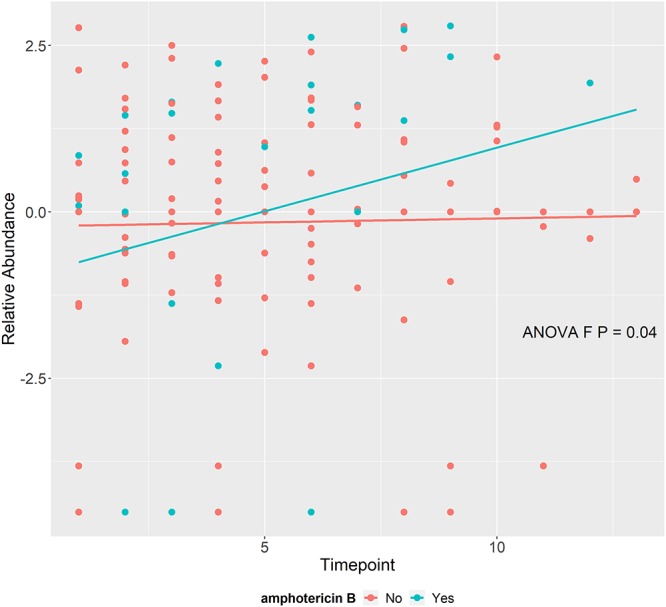
Changes in *Fusarium* over time stratified by amphotericin B administration. Centered log ratio (clr) data represent transformed relative abundances of *Fusarium* with fitted lines from the mixed-effects model over time according to whether amphotericin B was administered (aqua) or not (coral) during the study. *P* values were derived from the ANOVA F-test.

We next sought to determine if antimicrobial administration resulted in significant mycobiome changes during RIC for AML. We examined the three primary empirical broad-spectrum antibiotics received in this cohort, which were carbapenems, cephalosporins, and piperacillin-tazobactam, and found that 87% of the patients were administered at least one of these three broad-spectrum antimicrobials. Some patients received more than one broad-spectrum antibiotic prior to neutrophil recovery, with 67% receiving a carbapenem, 44% a cephalosporin, and 28% piperacillin-tazobactam. We also evaluated the three primary treatment antifungals, consisting of echinocandins, amphotericin B, and triazoles. All patients were administered at least one of these three antifungals, with 79% receiving an azole, 77% an echinocandin, and 15% amphotericin B. We analyzed the later time point during RIC (T6) given that all antimicrobials analyzed were administered by T6 and that it was the latest time point common among most patients. Surprisingly, there was no significant association between the relative abundance of the top 5 most abundant fungal taxa at time point 6 and receipt of specific antimicrobials (Tables S2 toS7). However, amphotericin B seemed to have a significant effect on the composition of the oral fungal community over time. Patients who received amphotericin B experienced an increase in the relative abundance of *Fusarium* over time (*P* = 0.034) (Fig. 5). There is biological plausibility to this observation inasmuch as *Fusarium* species are associated with high amphotericin B MICs (27). Amphotericin B had no significant effect on the relative abundance of other taxa over time.

10.1128/mSphere.00048-20.5 TABLE S2 Taxon abundance differences at T6 by receipt of piperacillin-tazobactam. *P* values represent results of Mann-Whitney test comparing the relative abundances of designated taxa between those who did and did not receive piperacillin-tazobactam. Listed are the top 5 taxa by abundance at T6; however, all genera were tested. Download Table S2, DOCX file, 0.01 MB. Copyright © 2020 Robinson et al. 2020 Robinson et al. This content is distributed under the terms of the Creative Commons Attribution 4.0 International license.

10.1128/mSphere.00048-20.6 TABLE S3 Taxon abundance differences at T6 by receipt of carbapenem. *P* values represent results of Mann-Whitney test comparing the relative abundances of designated taxa between those who did and did not receive a carbapenem. Listed are the top 5 taxa by abundance at T6; however, all genera were tested. Download Table S3, DOCX file, 0.01 MB. Copyright © 2020 Robinson et al. 2020 Robinson et al. This content is distributed under the terms of the Creative Commons Attribution 4.0 International license.

10.1128/mSphere.00048-20.7 TABLE S4 Taxon abundance differences at T6 by receipt of cephalosporin. *P* values represent results of Mann-Whitney test comparing the relative abundances of designated taxa between those who did and did not receive a cephalosporin. Listed are the top 5 taxa by abundance at T6; however, all genera were tested. Download Table S4, DOCX file, 0.01 MB. Copyright © 2020 Robinson et al. 2020 Robinson et al. This content is distributed under the terms of the Creative Commons Attribution 4.0 International license.

10.1128/mSphere.00048-20.8 TABLE S5 Taxon abundance differences at T6 by receipt of echinocandin. *P* values represent results of Mann-Whitney test comparing the relative abundances of designated taxa between those who did and did not receive an echinocandin. Listed are the top 5 taxa by abundance at T6; however, all genera were tested. Download Table S5, DOCX file, 0.01 MB. Copyright © 2020 Robinson et al. 2020 Robinson et al. This content is distributed under the terms of the Creative Commons Attribution 4.0 International license.

10.1128/mSphere.00048-20.9 TABLE S6 Taxon abundance differences at T6 by receipt of amphotericin B. *P* values represent results of Mann-Whitney test comparing the relative abundances of designated taxa between those who did and did not receive amphotericin B. Listed are the top 5 taxa by abundance at T6; however, all genera were tested. Download Table S6, DOCX file, 0.01 MB. Copyright © 2020 Robinson et al. 2020 Robinson et al. This content is distributed under the terms of the Creative Commons Attribution 4.0 International license.

10.1128/mSphere.00048-20.10 TABLE S7 Taxon abundance differences at T6 by receipt of triazole. *P* values represent results of Mann-Whitney test comparing the relative abundances of designated taxa between those who did and did not receive a triazole. Listed are the top 5 taxa by abundance at T6; however, all genera were tested. Download Table S7, DOCX file, 0.01 MB. Copyright © 2020 Robinson et al. 2020 Robinson et al. This content is distributed under the terms of the Creative Commons Attribution 4.0 International license.

### Mycobiome characteristics are correlated with bacterial infection outcomes.

**FIG 6:**
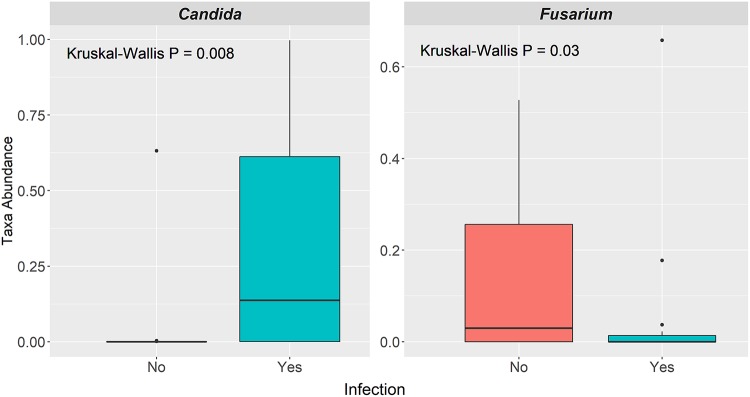
Relative abundance differences of taxa at individual time points among AML patients who did and did not experience bacterial infection during RIC. Box plots depict the taxon abundance differences of *Candida* at T3 and *Fusarium* at T6 between those patients that did (aqua) and did not (coral) contract bacterial infections prior to neutrophil recovery following RIC. *P* values are derived from Mann-Whitney test.

In light of the complex interplay between fungal populations and opportunistic bacterial pathogens (28), we sought to determine the relationships between the mycobiome and infections in AML patients during RIC. A total of 23% of patients had a microbiologically defined infection with a positive bacterial culture, which included mostly bacteremia or urinary tract infections (Table 1). We also considered the 31% of patients who had a clinically defined infection, such a pneumonia or cellulitis, in the absence of positive culture in our analysis. In analyzing the three major time points (T1, T3, and T6), we found that the relative abundance of *Candida* at T3 was significantly higher in patients that contracted microbiologically or clinically defined infections prior to neutrophil recovery (*P* = 0.008), while there was a significantly higher relative abundance of *Fusarium* (*P* = 0.03) at T6 in those that did not develop an infection prior to neutrophil recovery (Fig. 6). These results were corroborated using the LefSe (linear discriminant analysis of effect size) algorithm, where *Candida* was more differentially abundant at T3 among patients who developed an infection prior to neutrophil recovery (Fig. S2) and *Fusarium* more abundant at T6 among patients who did not have an infection (Fig. S3). No other statistical associations were observed for other genera at the three time points or at T1 (data not shown).

10.1128/mSphere.00048-20.2 FIG S2 Differential abundance of specific taxa at T3 for acute leukemia patients who had infectious complications during induction chemotherapy. The LefSe (linear discriminant analysis effect size) algorithm was used to determine which taxonomic units were most differentially abundant among acute leukemia patients who had infectious complications during induction chemotherapy (“1”) and those who did not experience infection (“0”) at T3. Download FIG S2, JPG file, 0.04 MB. Copyright © 2020 Robinson et al. 2020 Robinson et al. This content is distributed under the terms of the Creative Commons Attribution 4.0 International license.

10.1128/mSphere.00048-20.3 FIG S3 Differential abundance of specific taxa at T6 for acute leukemia patients who had infectious complications during induction chemotherapy. The LefSe (linear discriminant analysis effect size) algorithm was used to determine which taxonomic units were most differentially abundant among acute leukemia patients who had infectious complications during induction chemotherapy (“1”) and those who did not experience infection (“0”) at T6. Download FIG S3, JPG file, 0.04 MB. Copyright © 2020 Robinson et al. 2020 Robinson et al. This content is distributed under the terms of the Creative Commons Attribution 4.0 International license.

### Interkingdom interactions of the oral microbiome over time in AML patients receiving RIC.

**FIG 7:**
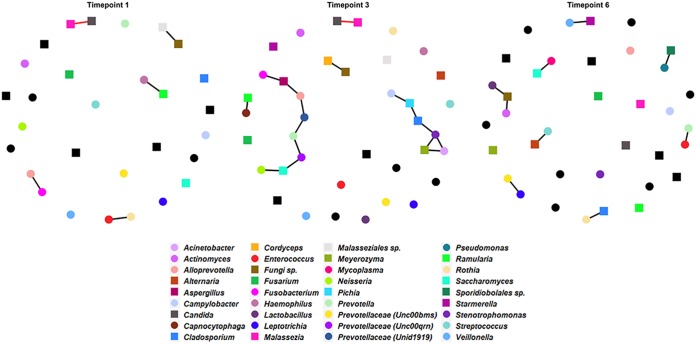
Cross-domain association networks across time. SPEIC-EASI networks were generated for time points 1, 3, and 6. Circles indicate bacterial taxa, while squares indicate fungal taxa. Cooccurrence (positive) relationships are indicated with black edges. Coexclusion (negative) relationships are indicated with red edges. Colors are indicated for each taxon in the inlaid legend and are constant across networks. Nodes that are black have no connection in any network.

Cross-domain association networks were constructed at T1, T3, and T6 using matching bacterial and fungal microbiome samples (*n* = 272). The three SPIEC-EASI (sparse inverse covariance estimation for ecological association inference) networks show vast differences in intra- and interkingdom connectivity between time points, highlighting the dynamic nature of bacterial and fungal interactions induced by changes in the community during RIC (Fig. 7). The average node degrees were 0.43 (standard deviation [SD], ±0.48), 0.84 (SD, ±0.92), and 0.5 (SD, ±0.56) for T1, T3, and T6, respectively.

At baseline (T1), we observed one cross-domain connection (*Ramularia* with *Haemophilus*), two bacterial connections (*Fusobacterium* with *Alloprevotella* and *Enterococcus* with *Rothia*), and two fungal connections (*Malassezia* with *Candida* and “Fungi sp.” [unknown/unidentified fungal genus] with Malasseziales sp.). The T3 network contained nine cross-domain connections (*Cladosporium* with *Stenotrophomonas*, *Aspergillus* with *Fusobacterium*, *Aspergillus* with *Alloprevotella*, *Saccharomyces* with *Prevotellaceae* [*Unc00qrn*], *Saccharomyces* with *Neisseria*, *Meyerozyma* with Acinetobacter, *Meyerozyma* with *Stenotrophomonas*, *Pichia* with *Campylobacter*, and *Ramularia* with *Capnocytophaga*), four bacterial connections (Acinetobacter with *Stenotrophomonas*, *Prevotellaceae* [*Unc00qrn*] with *Prevotella*, *Alloprevotella* with *Prevotellaceae* [*Unid1919*], and *Prevotellaceae* [*Unid1919*] with *Prevotella*), and three fungal connections (*Cladosporium* with *Pichia*, *Cordyceps* with *Fungi* sp., and *Candida* with *Malassezia*). At the latest time point analyzed (T6), we observed seven cross-domain connections (*Alternaria* with *Streptococcus*, *Cladosporium* with *Rothia*, *Saccharomyces* with *Mycoplasma*, *Sporidiobolales* sp. with *Pseudomonas*, *Fungi* sp. with *Lactobacillus*, *Fungi* sp. with *Actinomyces*, and *Starmerella* with *Veillonella*) and two bacterial connections (*Enterococcus* with *Prevotella* and *Prevotellaceae* [*Unc00bms*] with *Leptotrichia*).

Some of the intrakingdom and interkingdom interactions have been previously observed. Specifically, *Malassezia* and *Candida* are often coisolated from infectious samples from the skin infections (29) and onychomycosis (30), but to our knowledge cooccurrence has not been shown in the oral cavity. Interestingly, we found a negative correlation between *Malassezia* and *Candida* at T1 and T3 in the oral cavity. In a study of the oral microbiome of HIV patients versus uninfected individuals (31), it was found that *Cladosporidium* and *Rothia* had a positive correlation in uninfected individuals. We observed the same cooccurrence at T6 in treated leukemia patients. Also, in individuals uninfected with HIV, there was a negative correlation between *Aspergillus* and *Prevotella* (31). We observed the same cross-domain connection, but as a cooccurrence. In the same study, the authors observed cocolonization of *Cladosporidium* and *Pichia* in uninfected individuals (31), which is a fungal relationship that we detected at T3. Additionally, we saw a bacterial cooccurrence relationship between *Prevotellaceae* and *Leptotrichia* at T6, which has also been observed in subjects with apical periodontitis (32) and in subjects with halitosis, as well as in healthy individuals (33).

Only one connection overlapped any two networks. Interestingly however, time point 3 showed more-dependent connectivity (multiple nodes linked to each other), while time points 3 and 6 had increased connectivity overall. As we had previously reported loss of bacterial diversity over time among the patients included in these analyses (2), this suggests that either loss of bacterial diversity or changes in the oral environment under conditions of chemotherapeutic and antimicrobial pressure or both affect the bacterium-fungus interplay. This may potentially result in clinical consequences, as organisms that do not typically interact would inhabit the niche. This could potentially have acute consequences for functional metabolites, genetic exchange, and alterations in the virulence of the pathogen or in the immune status of the host (28, 34).

### Limitations.

Some fungus-bacterium interactions in the oral cavity of diseased and healthy individuals (namely, *Candida*-bacterium interactions) have been intensely studied; however, interactions of bacteria with other known fungal inhabitants of the oral cavity have yet to be described. Thus, the impact of fungus-bacterium interactions in the oral cavity and on overall health is not completely understood. Although this exploratory study had a relatively small sample size and assessed only oral samples, the data shown here still highlight the dynamic nature of mycobiome and bacteriome communities and their interactions over the course of RIC. Unfortunately, the study design did not include information on diet, the metabolic milieu (e.g., other relevant comorbidities such as diabetes), oral health information (including oropharyngeal candidiasis), or occurrence of mucositis. These clinical factors/outcomes could potentially account for the heterogeneity seen within the data or might also be associated with specific mycobiome or bacteriome compositions/relationships. Despite not including a comprehensive record of clinical covariates, we were able to determine that specific changes in the mycobiome community structure appear to have clinical consequences in terms of bacterial infections. Moreover, interkingdom dynamics are likely driven by changes in the mycobiome due to chemotherapy intensity and subtype, as well as bacteriome changes driven by antibiotic exposures. Although this report highlights the underappreciated complex and dynamic nature of the cross-kingdom interactions that occur over time and result from the stress of chemotherapy and antibiotic therapy, these data are limited by our having used only marker gene analysis (16S rRNA gene and internal transcribed spacer 2 [ITS2] sequencing) to identify bacterium-fungus dynamics. Target gene analysis is limited in its ability to differentiate individual taxa, specifically to lower taxonomic levels such as the species level. Although the ability to assemble and differentiate fungal reads at the whole-genome level is currently restricted by the number of fungal genomes available in public databases, this information could be vastly improved in the future via shotgun metagenomics sequencing, particularly in regard to functional pathway analysis. An alternative approach would be to layer on metabolomics or metaproteomics to understand the functional consequences of bacterial and fungal changes as well as the interkingdom metabolic pathway interactions. Again, however, methods for data integration of shotgun metagenomic and metabolomics data, particularly in regard to cross-domain networks, are still in their infancy.

### Conclusions.

Here, we provide the first longitudinal analysis of the oral mycobiome in patients with acute leukemia combined with parallel oral bacteriome data. Incorporation of fungal analyses into microbiome studies may help improve mechanistic understanding of how commensal flora impact clinical outcomes. These data suggest that simultaneous mycobiome and bacteriome longitudinal analyses may add additional insight in order to fully understand how the microbiome impacts infectious risk in immunocompromised patients. We anticipate these data will inspire future studies aimed to further elucidate these relationships in the cancer treatment setting.

## MATERIALS AND METHODS

### Patient sample collection, DNA extraction, sequencing, and bioinformatics.

In this pilot exploratory study, we collected oral (buccal swab) samples (*n* = 299) from 39 newly diagnosed adult AML patients undergoing IC at the MD Anderson Cancer Center (MDACC) in Houston, TX, from September 2013 to August 2015. These oral samples and accompanying 16S rRNA gene sequences were derived from a previously reported cohort (2). The study protocol was approved by the MDACC Institutional Review Board (PA13-0339), and the study was conducted in compliance with the Declaration of Helsinki. Written informed consent was obtained from all participants before enrollment. Buccal swabs were collected by swabbing the inside the cheek 3 times on each side using a Catch-All sample collection swab (Epicentre), and the swab tips were then placed in sterile 2-ml cryovials and maintained at −80°C until DNA extraction. Collection of samples from each patient was performed at baseline, continued approximately every 96 h, and stopped upon neutrophil recovery. Clinical data, including antibiotic and antifungal administration, chemotherapy subtypes, and infection status, were extracted from the electronic medical records. Infections prior to neutrophil recovery were classified as microbiologically defined infections (MDI) or clinically defined infections (CDI) using established guidelines (35). Patients were categorized as receiving (i) non-fludarabine-based high-intensity regimens, (ii) high-intensity fludarabine-containing regimens, (iii) low-intensity hypomethylating-agent-based therapy, or (iv) other low-intensity/low-dose cytarabine-based regimens.

Microbial DNA was extracted from oral swabs using the MO BIO PowerSoil DNA isolation kit protocol as described previously (36). We performed ITS sequencing on a subset of patients (the first sequential 39 patients enrolled on study with available residual sample) in which we had previously characterized the microbiome using 16S rRNA gene sequencing (2, 37–39). The internal transcribed spacer 2 (ITS2) region was amplified from oral swab DNA using primers ITS3 and ITS4 as described previously (36). Each primer included an Illumina adapter and linker sequence, and the reverse primer (ITS4) also contained a unique 12-bp Golay barcode (40). Amplicons were sequenced by the use of an Illumina MiSeq platform with the 2 × 300-bp paired-end protocol. The ITS2 read pairs were demultiplexed based on the unique barcodes. Reads were merged and filtered using default settings in USEARCH v7.0.1090 (41). ITS2 sequences were clustered into operational taxonomic units (OTUs) at a similarity cutoff of 97% using the UPARSE pipeline (42). Chimeras were removed using USEARCH v8.0.1517 and UCHIME (43). OTUs were aligned against sequences from the NCBI GenBank Plant and UNITE databases (44). Abundance data were recovered by mapping the demultiplexed reads to the UPARSE OTUs. A custom script developed at the Center for Metagenomics and Microbiome Research at Baylor College of Medicine was used to construct an OTU table. Unmapped (<80% identity or <95% coverage) OTUs were manually analyzed by BLASTN (37). After we performed filtering that included only the samples which had corresponding clinical and 16S data to match the ITS data, 290 samples were obtained for further analyses.

### Statistical methods.

For β-diversity, principal-coordinate analysis (PCoA) plots were produced using Bray-Curtis distances. A mixed-effects model was applied to determine if time was a significant predictor of α-diversity. A random intercept for patients was included. A Kruskal-Wallis test was performed to compare α-diversity and taxon abundance data between chemotherapy subtype groups. In association testing, we focused on the top five most abundant fungal genera based on average abundance: *Candida*, *Cladosporium*, *Fusarium*, *Malassezia*, and *Saccharomyces*. Similarly, a Mann-Whitney test was performed to compare both alpha-diversity data and taxon abundance data corresponding to chemotherapy intensity (high versus low), use of amphotericin B (yes versus no), and infection status (yes versus no). The linear discriminant analysis effect size (LEfSe) algorithm was used to test whether taxon abundance predicted infection status. The LEfSe algorithm was run using the Galaxy module with default settings. Mixed-effects models were used to determine whether taxon abundance significantly changed over time. Analyses based on these models were performed on the centered log ratio (clr) transformed relative abundance data to address the compositional characteristics of the data. Each model contained the following variables: time point, the variable of interest, and an interaction between the two. To account for repeated measures, a random intercept for patient was included in the model. An analysis of variance (ANOVA) F-test was applied to determine whether time and the variable of interest were significantly associated. The statistical analyses performed as a part of this study were exploratory in nature. In general, *P* values of less than 0.05 were considered significant; for analyses across the mycobiome, we computed false-discovery-rate (FDR)-adjusted *P* values, which are provided in the supplemental tables. Data analysis and visualization were performed using R, mainly using functions from the R packages ggplot2 and phyloseq (26, 45).

### Cross-domain association networks.

SPIEC-EASI was used to construct the cross-domain microbial networks at the genus level for both bacteria and fungi. For the tables listing bacterial and fungal genera, the data corresponding to time points 1 (T1), 3 (T3), and 6 (T6) were separated (46). As statistically sound methods for longitudinal analyses/trends are underdeveloped for microbiome network analyses, we aimed at analyzing samples at baseline prior to chemotherapy and antimicrobial administration (T1), at a time point after chemotherapy administration (T3), and at a time point after antimicrobial administration (T6). As we had significant sample dropout after T6, and given our already small sample size, T6 was the last time point which retained most samples. In addition, all patients who received broad-spectrum antibiotics had received at least one type of broad-spectrum antibiotic by T6. For each OTU table, taxa are included in the network analysis if the mean abundance was greater than 0.01. T1 had 35 samples with 16 bacterial taxa and 13 fungal taxa. T3 was represented by 36 samples, with 22 bacterial taxa and 16 fungal taxa. T6 contained 31 samples with 21 bacterial taxa and 15 fungal taxa. The sparsity parameter λ was set to 0.3, and the value corresponding to λ was set to 50. We verified the stability of our results for a single value of λ. We also examined sensitivity across multiple λ values and found that the networks were not sensitive to the choice of λ.

### Data availability.

All 16S rRNA gene sequences were deposited in the NCBI Sequence Read Archive (https://www.ncbi.nlm.nih.gov/sra) under BioProject identifiers (IDs) PRJNA352060 and PRJNA526551, whereas ITS2 sequences were submitted under the BioProject ID PRJNA599151 (the authors could not make this BioProject record available at the time of this paper’s publication due to circumstances related to the COVID-19 pandemic, but it will be made accessible as soon as possible after publication).
