# Supplementary figures and images for: Observational Cohort Study of Oral Mycobiome and Interkingdom Interactions over the Course of Induction Therapy for Leukemia
Source: mSphere. 2020 Apr 15;5(2):e00048-20. doi: 10.1128/mSphere.00048-20 (PMC7160678; doi:10.1128/mSphere.00048-20)

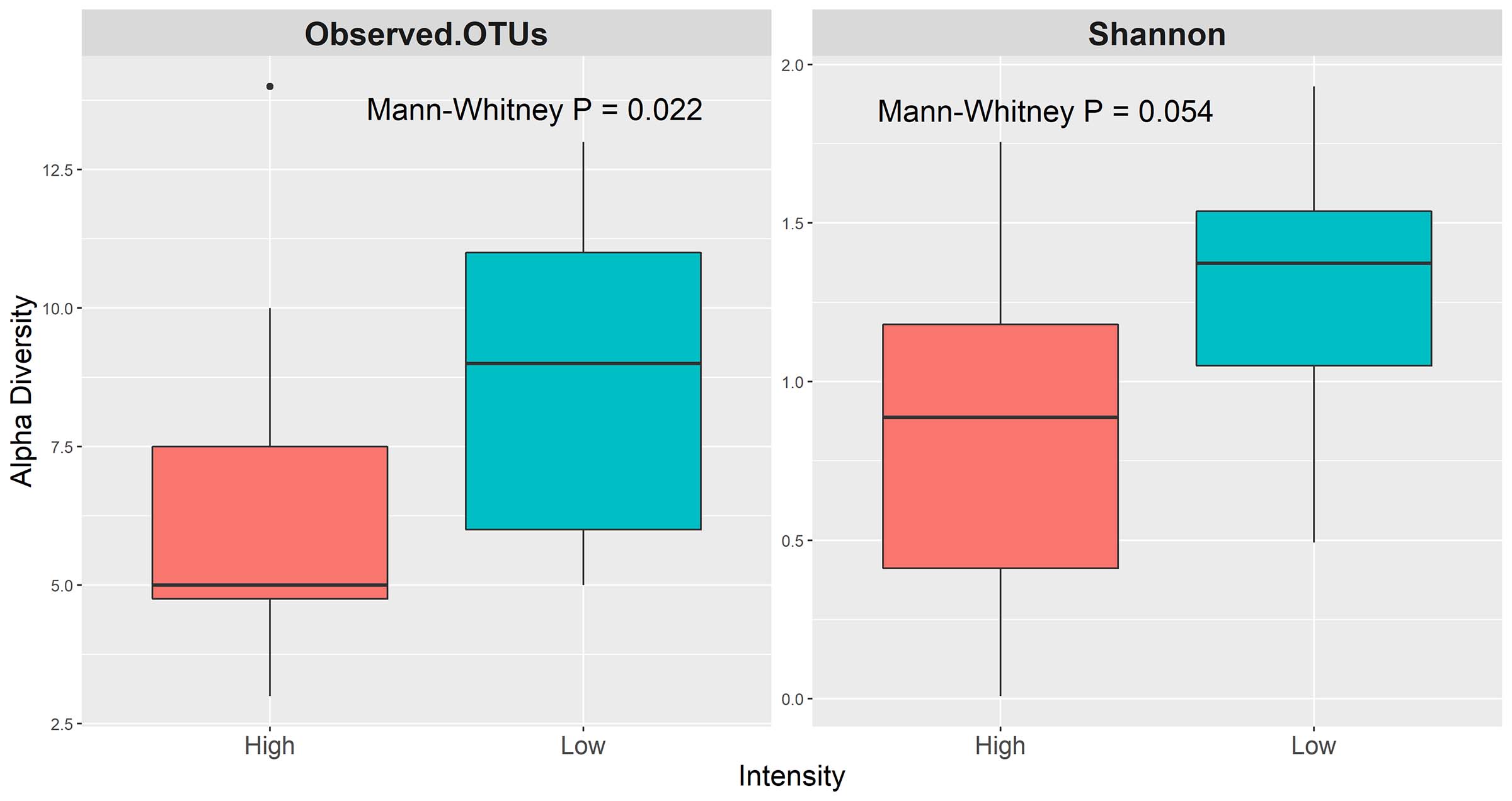

Supplement: FIG S1 [file mSphere.00048-20-sf001.jpg]

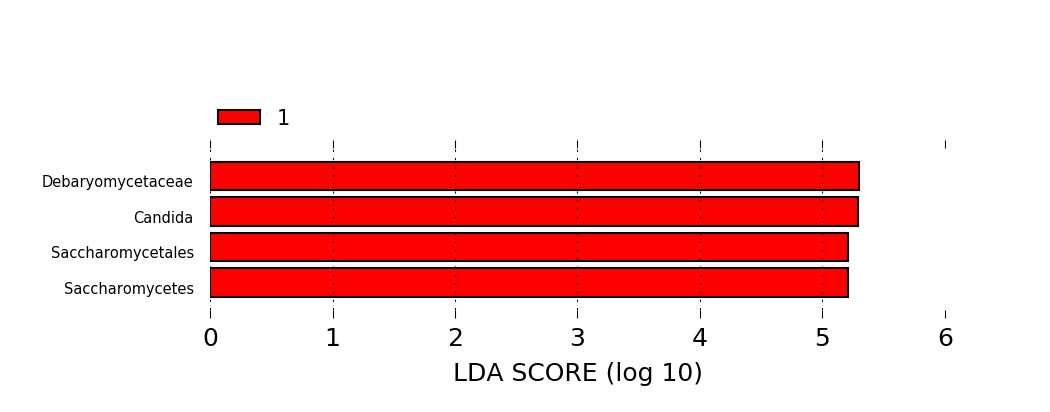

Supplement: FIG S2 [file mSphere.00048-20-sf002.jpg]

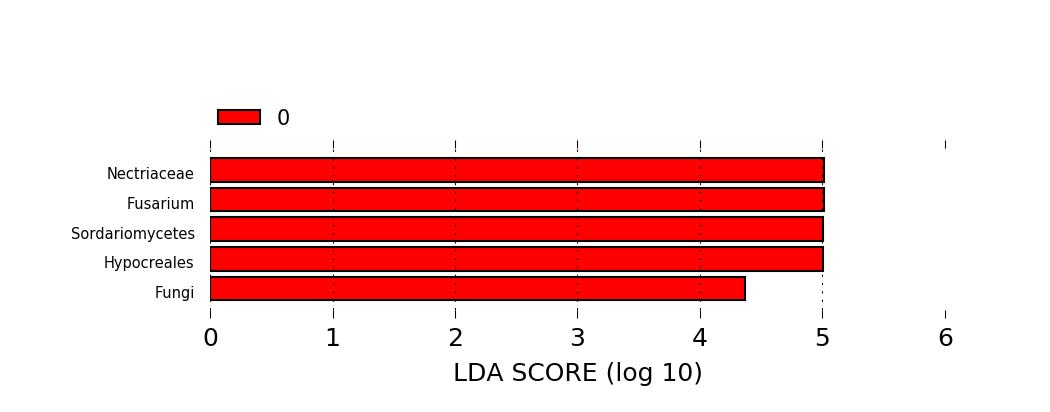

Supplement: FIG S3 [file mSphere.00048-20-sf003.jpg]
