# Supplementary material for: Observational Cohort Study of Oral Mycobiome and Interkingdom Interactions over the Course of Induction Therapy for Leukemia
Source: mSphere. 2020 Apr 15;5(2):e00048-20. doi: 10.1128/mSphere.00048-20 (PMC7160678; doi:10.1128/mSphere.00048-20)
Supplement: TABLE S1 [file mSphere.00048-20-st001.docx]

| Taxon | Time 1 | Time 2 | Time 3 | Time 4 | Time 5 | Time 6 |
| --- | --- | --- | --- | --- | --- | --- |
| *Fusarium* | 0.838 | 0.476 | 0.605 | 0.222 | 0.907 | 0.689 |
| *Candida* | 0.314 | 0.360 | 0.115 | 0.526 | 0.972 | 0.249 |
| *Saccharomyces* | 0.541 | 0.724 | 0.621 | 0.814 | 0.803 | 0.718 |
| *Malassezia* | 0.809 | 0.751 | 0.311 | 0.929 | 0.342 | 0.214 |
| *Cladosporium* | 0.289 | 0.318 | 0.172 | 0.145 | 0.147 | 0.954 |

^a^ Subtypes are fludarabine-containing high intensity regimens, non-fludarabine containing high intensity regimens, hypomethylators, and “others”.
