# Supplementary material for: Observational Cohort Study of Oral Mycobiome and Interkingdom Interactions over the Course of Induction Therapy for Leukemia
Source: mSphere. 2020 Apr 15;5(2):e00048-20. doi: 10.1128/mSphere.00048-20 (PMC7160678; doi:10.1128/mSphere.00048-20)
Supplement: TABLE S4 [file mSphere.00048-20-st004.docx]

| Taxa | P-Value | FDR-Adj. P |
| --- | --- | --- |
| *Candida* | 0.43 | 0.60 |
| *Cladosporium* | 0.59 | 0.74 |
| *Fusarium* | 0.36 | 0.56 |
| *Malassezia* | 0.76 | 0.92 |
| *Saccharomyces* | 0.05 | 0.56 |
