# Supplementary material for: Observational Cohort Study of Oral Mycobiome and Interkingdom Interactions over the Course of Induction Therapy for Leukemia
Source: mSphere. 2020 Apr 15;5(2):e00048-20. doi: 10.1128/mSphere.00048-20 (PMC7160678; doi:10.1128/mSphere.00048-20)
Supplement: TABLE S7 [file mSphere.00048-20-st007.docx]

| Taxa | P-Value | FDR-Adj. P |
| --- | --- | --- |
| *Candida* | 0.07 | 0.37 |
| *Cladosporium* | 0.75 | 0.91 |
| *Fusarium* | 0.77 | 0.91 |
| *Malassezia* | 0.76 | 0.91 |
| *Saccharomyces* | 0.91 | 0.91 |
